# Advancements in 3D spheroid imaging: Optimised cryosectioning and immunostaining techniques

**DOI:** 10.1016/j.mex.2023.102415

**Published:** 2023-10-06

**Authors:** Claire Charlet-Faure, Annemette Præstegaard Thulesen, Adelina Rogowska-Wrzesinska

**Affiliations:** a Department of Biochemistry and Molecular Biology, University of Southern Denmark, Campusvej 55, Odense M DK-5230, Denmark; b Faculté des Sciences Site St Charles Aix Marseille Université, 3 place Victor Hugo, Marseille 13331, France

**Keywords:** Antigen retrieval and cryosectioning of multicellular 3D spheroids, Spheroids, Organoids, Antigen retrieval, Cryosectioning, Immunostaining, Fluorescent microscopy

## Abstract

This article presents a modified protocol for embedding and sectioning spheroids and organoids, which are increasingly used in research due to their ability to emulate living tissue. The modifications aim to reduce the distortion and damage of these fragile structures during the embedding and sectioning process. The new method involves using optimized embedding containers, a modified embedding protocol, and optimized temperatures for cryosectioning. A heat-induced antigen retrieval protocol was tested and found to significantly increase immunostaining intensity without compromising spheroid integrity. The combined approach allowed for the creation of thinner cryosections, leading to clearer and more detailed images. The results suggest that the modified protocol could be widely adopted to enhance the imaging of spheroids and organoids.

• Paraformaldehyde fixation of spheroids
• Antigen retrieval treatment of spheroids
• Embedding in freezing medium and cryosectioning

## Graphical abstract

**Figure:**
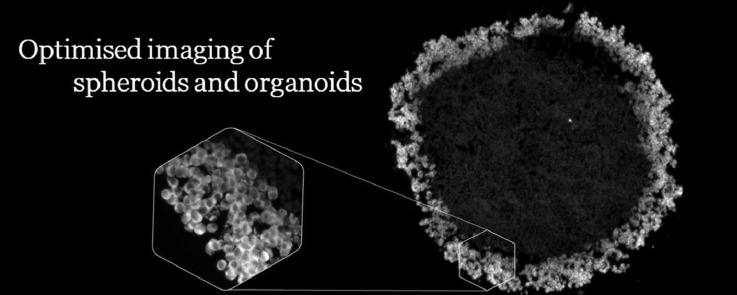

Specifications Table

**Table:** 

Subject area:	Biochemistry, Genetics and Molecular Biology
More specific subject area:	Cell biology, tissue engineering
Name of your method:	Antigen retrieval and cryosectioning of multicellular 3D spheroids
Name and reference of original method:	K. Wrzesinski, H.S. Frandsen, C. Calitz, C. Gouws, B. Korzeniowska, S.J. Fey, Clinostat 3D Cell Culture: Protocols for the Preparation and Functional Analysis of Highly Reproducible, Large, Uniform Spheroids and Organoids, Methods Mol Biol 2273 (2021) 17–62
Resource availability:	Antigen Retrieval: https://youtu.be/FpAahm_o3GQ Embedding spheroids: https://youtu.be/kv_0rUQjpo0 Cryosectioning: https://youtu.be/o_FFdVFJ2PQ

## Method details

### Background

Spheroids and organoids, which are three-dimensional cell culture structures that emulate the architecture of living tissue, are becoming increasingly popular in research. They have the potential to provide insights into various biological processes while reducing the need for animal experimentation [1].

Spheroids must be cut into 10 to 30 µm slices to observe their inner structure. The most common method is embedding spheroids in a freezing medium to obtain a solid block that can be cut using a cryotome [2]. This approach was originally developed for sectioning animal and plant tissues, and the existing protocols are not optimal for handling small and fragile spheroids and organoids. Typically, spheroids larger than 600 µm in diameter develop a distinctly structured necrotic core [3]. The necrotic core is located in the central part of the spheroids and consists of compacted cell debris and DNA fragments. That causes the structures to be more fragile during cryosectioning and results in a distinct staining pattern between the rim (the outer layer of the spheroid) and the core.

To prevent distortions and damage to the spheroids structure, we tested and optimized the embedding method by using new embedding moulds that allowed us to precisely place the spheroids in the freezing medium, reduce the transfer and handling of liquids, as well as the reduction of air bubble formation around the spheroids, the modifications introduced in this protocol are summarised in Table 1. This new approach permitted the creation of thinner blocks with a triangle shape that promoted precise cuts with minimal loss of specimen material. Additionally, we optimized the temperatures used for the cryosectioning and the procedure for collecting sectioned spheroids on the microscopy glass slides. Increasing the temperature of the specimen (−13 °C) produced sections of greater elasticity that adhered to glass slides more evenly.

**Table 1:** Summary of the optimized protocol's key steps compared to the original one.

Protocol step	Original	Optimized
Fixative	Formaldehyde solution 37 wt.% in H_2_O, 10–15% methanol	4% (w/v) paraformaldehyde in PBS, pH 7.3, freshly prepared
Fixation (min)	30	30 to 120
Antigen retrieval	No	Yes
Mould	Eppendorf tube	Tissue-Tek® Cryomold
Cryobar temp.	−50 °C	−50 °C
Chamber temp.	N/A	−24 °C
Specimen temp.	N/A	−13 °C

We also tested an antigen retrieval protocol that can be used to improve the accessibility of the antigens in the sample. That can result in greater fluorescence intensity after the immunostaining process. We chose to perform heat-induced antigen retrieval over an enzymatic process because the method is more aggressive, which might cause structural damage to the sample. The antigen retrieval procedure was performed on intact spheroids before embedding them in the freezing medium to reduce the risk of damage and loss of samples [4]. Different immersion times were tested to optimize the retrieval effect and procedure handling. We tested an antigen retrieval solution based on a sodium-citrate buffer at pH 6.0. We anticipate the procedure will have a similar effect when used with other retrieval buffers.

Modifications described in this protocol allowed us to efficiently obtain spheroids sections with preserved overall structure and little cell damage. Heat-induced antigen retrieval resulted in a significant amelioration of the antibody staining intensity. All the information needed to reproduce the method can be found in this article and the related videos.

### Preparation of spheroids

Spheroids were prepared according to a previously published protocol [2] using HepG2/C3A cells. Protocols described here were tested using 21-day-old spheroids with a diameter between 800 and 900 µm.

### Fixation

Spheroids were fixed in freshly prepared, 4% (w/v) paraformaldehyde in PBS, pH 7.3, for 2 h at room temperature. Note that treatment with freshly prepared, 4% (w/v) paraformaldehyde in PBS, pH 7.3 resulted in higher observable fluorescent intensity as compared to samples treated with formaldehyde solution 37 wt.% in H_2_O, stabilized with 10–15% methanol, (Fig. 5). Shorter (30 min) and longer (up to 2 weeks) fixation intervals were also tested. Prolonged exposure to paraformaldehyde resulted in decreased signal, Fig. 5, which is most likely related to excessive cross-linking of the proteins and loss of antigen epitopes.

### Antigen Retrieval

A video of the method is available at: https://youtu.be/FpAahm_o3GQ.

#### Reagents

• Sodium Citrate Buffer (10 mM Sodium Citrate, 0.05% Tween 20, pH 6.0) [5], Note 3.1.
• 30% sucrose.
• PBS.

#### Equipment

• Heat-resistant basket.
• Water bath at 92–95 °C.

#### Procedure

1. Immerse fixed spheroids in 200 µl of the retrieval solution for at least 8 h or overnight at 4 °C, Note 3.2.
2. Preheat the retrieval solution's fresh portion (200 µl) to 92–95 °C.
3. Replace the cold retrieval solution with 1 ml of the fresh, preheated one. Incubate at 92–95 °C for 10 min, Note 3.3.
4. Remove the retrieval solution.
5. Add 1 ml of 30% cold (4 °C) sucrose solution.
6. Incubate at 4 °C for two hours.

Note 3.1. It may be beneficial to utilize buffers with a distinct pH value and ionic strength, optimal for retrieving the desired antigen [6].

Note 3.2 Moving the liquids rather than the spheroids is recommended to minimize handling damage to the spheroids.

Note 3.3 It is advisable to adjust the heating time based on the size of the spheroid. For example, a smaller specimen may benefit from a shorter incubation time.

### Embedding spheroids in freezing medium:

A video of the procedure is available at: https://youtu.be/kv_0rUQjpo0.

#### Reagents

• Tissue freezing medium, PolyFreeze, Sigma-Aldrich® (Cat. nr. SHH0025, Merck), Note 4.1.

#### Equipment

• Standard Tissue-Tek® Cryomolds®, 20 × 25 mm, 5 mm height (Sakura® Finetek, Cat. No.4557).
• Aluminium foil.
• Dry ice (or isopentane cooled to -55 °C using a liquid nitrogen bath).

#### Procedure

1. To create a 3 x 35 mm thin band, repeatedly fold and compress a piece of aluminium foil.
2. Add a 1–2 mm layer of freezing medium at the bottom of the disposable cryomould.
3. Place the aluminium band diagonally in the mould to separate it into two triangles, Fig. 1A.

**Fig. 1:**
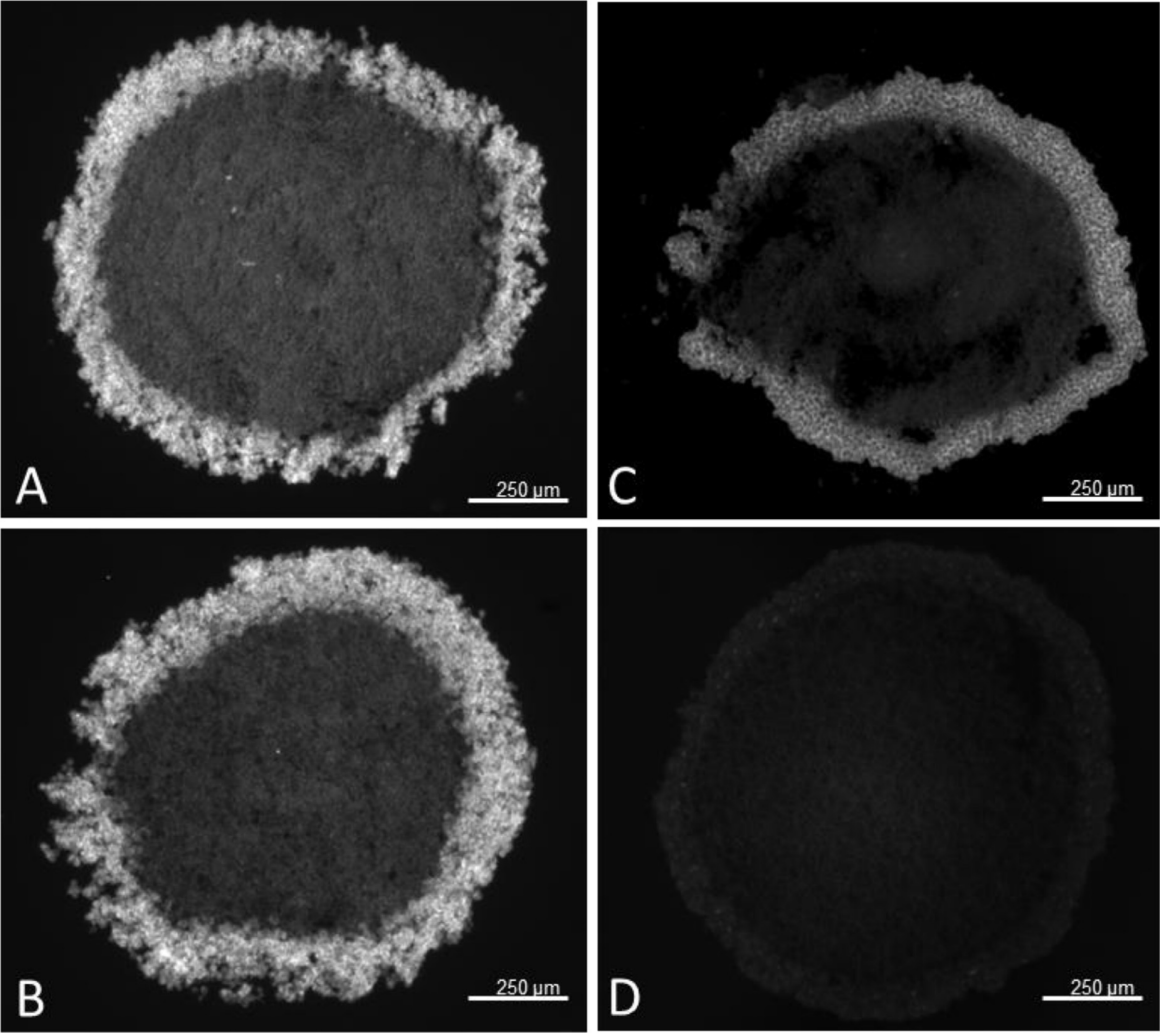
Effect of paraformaldehyde treatment on 30 µm sections of 21-day-old HepG2/C3A spheroids stained for α-SMA. (A) Two hours in fresh paraformaldehyde, (B) Two weeks in fresh paraformaldehyde, (C) Two hours in stabilized paraformaldehyde, (D) Two weeks in stabilized paraformaldehyde. All images were captured using a 4x objective.
4. Place the spheroids on top of the freezing medium. Ensure that the spheroids do not touch each other or the walls of the mould, Fig. 1A.
5. Remove any liquid transferred together with the spheroids.
6. Cover the spheroids with a small amount of freezing medium, do not overfill the mould.
7. Use an additional empty mould to gently apply pressure to the top of the freezing medium-filled mould. This process aids in the elimination of air bubbles and ensures that the embedding block is uniformly formed.
8. Invert the specimen to allow the spheroids to detach from the mould's bottom and migrate towards the freezing medium's central part.
9. Freeze the spheroids by placing the whole specimen on dry ice as soon as the spheroids are not touching any of the mould's walls.
10. Wait 5–10 min to let the freezing medium solidify.
11. Store the frozen tissue blocks at -80 °C.

Note 4.1 Incorporating coloured tissue freezing medium into the experimental procedure can facilitate the detection of spheroids during both the cutting and embedding processes.

### Cryosectioning

Video of the method: https://youtu.be/o_FFdVFJ2PQ.

#### Reagents

• Tissue freezing medium, PolyFreeze, Sigma-Aldrich® (Cat. nr. SHH0025, Merck).

#### Equipment

• Dry ice (or isopentane cooled to -55 °C using a liquid nitrogen bath).
• Slide box.
• SuperFrost glass slides (Cat. nr. 631–0108, VWR).
• Tweezers.
• Shandon® Cryotome FE and FSE, Thermo Fisher Scientific (A78910100).

#### Procedure

1. Turn on the cryotome one hour before starting cryosectioning. Set the following temperatures: Cryobar -50 °C, Chamber -24 °C, Specimen -13 °C, Note 5.1.
2. Prepare dry ice for the storage of samples and the slide box.
3. Place the specimen in the cryotome chamber to prevent thawing.
4. Remove the sample from the plastic mould and separate the freezing medium square into two triangles at the site where the aluminium foil was placed.
5. Apply freezing medium onto the cryocassette and attach the sample with the triangle's base facing downwards, Fig. 1B, Note 5.2.
6. Put the cryocassette onto the cryobar and let the freezing medium solidify.
7. Place the cryocassette in the quick-release clamp on the specimen head, Fig. 1B, Note 5.3.
8. Adjust the distance on the control panel so that the sample is close to the blade.
9. Adjust the cryotome knife to obtain the desired slice thickness. For fluorescent microscopy, slices between 5 and 30 µm thick are recommended.
10. Cut the sample until a spheroid structure emerges.
11. Collect the slices containing the desired spheroid structures by placing them with a tweezer on a cold glass slide, Note 5.4 and 5.5.
12. Apply gentle pressure at the bottom side of the glass slide using a fingertip. That will encourage the cryosectioned slice to adhere smoothly to the glass slide without forming air bubbles.
13. While cutting, keep the glass slides frozen; Note 5.6.
14. Check if the desired spheroid structures are present using a light microscope.
15. Store the glass slides at -80 °C.

Note 5.1 The temperatures of the specimen and the chamber will affect the plasticity of the obtained tissue sections and should be optimized for each type of sample, cryotome and freezing medium.

Note 5.2 Placing the specimen with the triangle base facing the cryocassette improves its adhesion to the cryocassette.

Note 5.3 Placement with the thinnest section of the triangle facing the cutting blade promotes smooth specimen cuts.

Note 5.4 The glass slides and the tweezer must be frozen so the sample slice does not melt when handled. We recommend keeping the glass slides and the tweezer inside the cryostat or on the cryobar.

Note 5.5 Collect consecutive slices from each spheroid to ensure that slices representing the central part of the spheroid are collected.

Note 5.6 Five to six slices can be placed securely on a standard microscopy slide.

### Immunostaining

Proteins in the cryosectioned spheroids were visualized according to a previously published protocol [2,7] using Recombinant Alexa Fluor® 488 Anti-alpha smooth muscle Actin antibody [SP171] (ab267536).

#### Microscopy

Images of the spheroids were taken with IX81 Motorized Inverted System Microscope (Olympus Corporation) equipped with LED fluorescent light source and ORCA-spark Digital CMOS camera (Hamamatsu Photonics K.K.) The following objectives have been used for obtaining images presented in this manuscript: Olympus 4x UPlanFl PhL / Phase Contrast Objective N.A.: 0.13 and Olympus 20x LCAch Infinity-Corrected PhC Phase Contrast Objective N.A.: 0.40.

#### Method validation

**Fig. 2:**
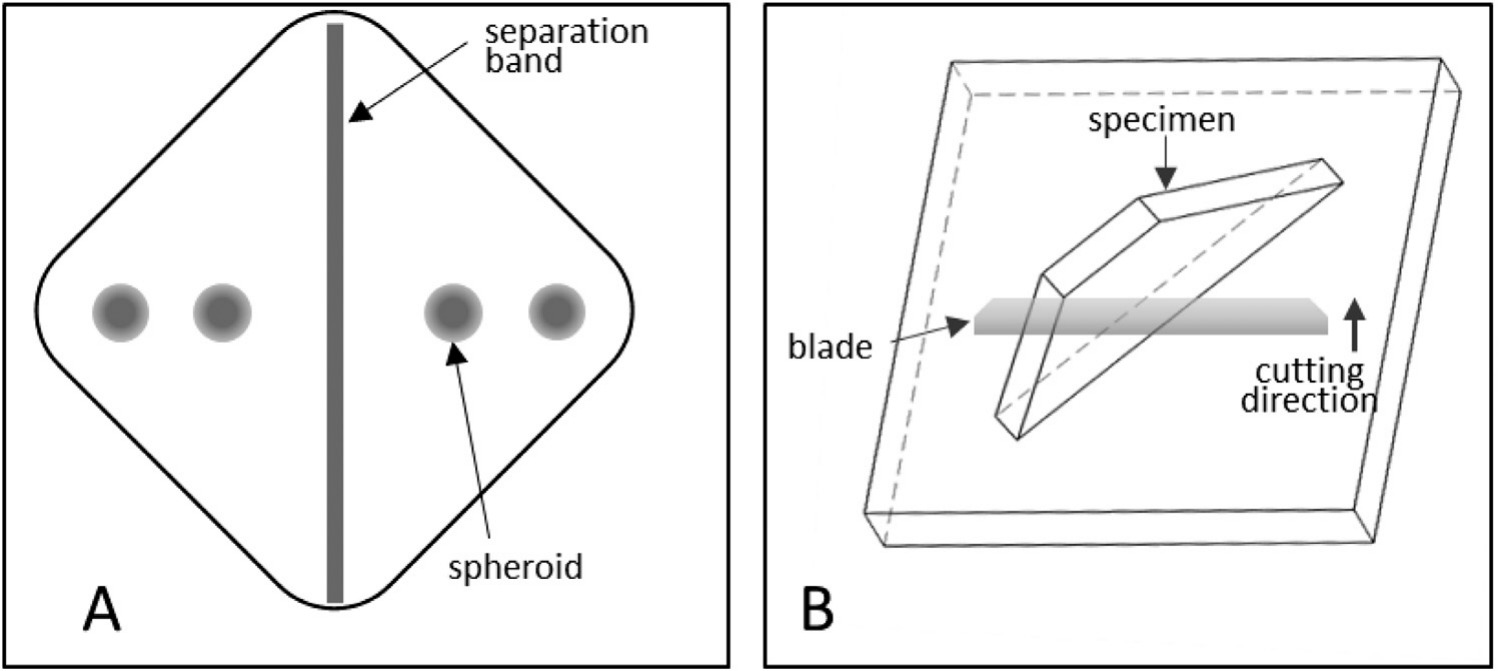
Preparation of embedded specimen. (A) Optimal positioning of spheroids and the separation band in the embedding mould. (B) Recommended specimen orientation facilitating optimal sectioning.

**Fig. 3:**
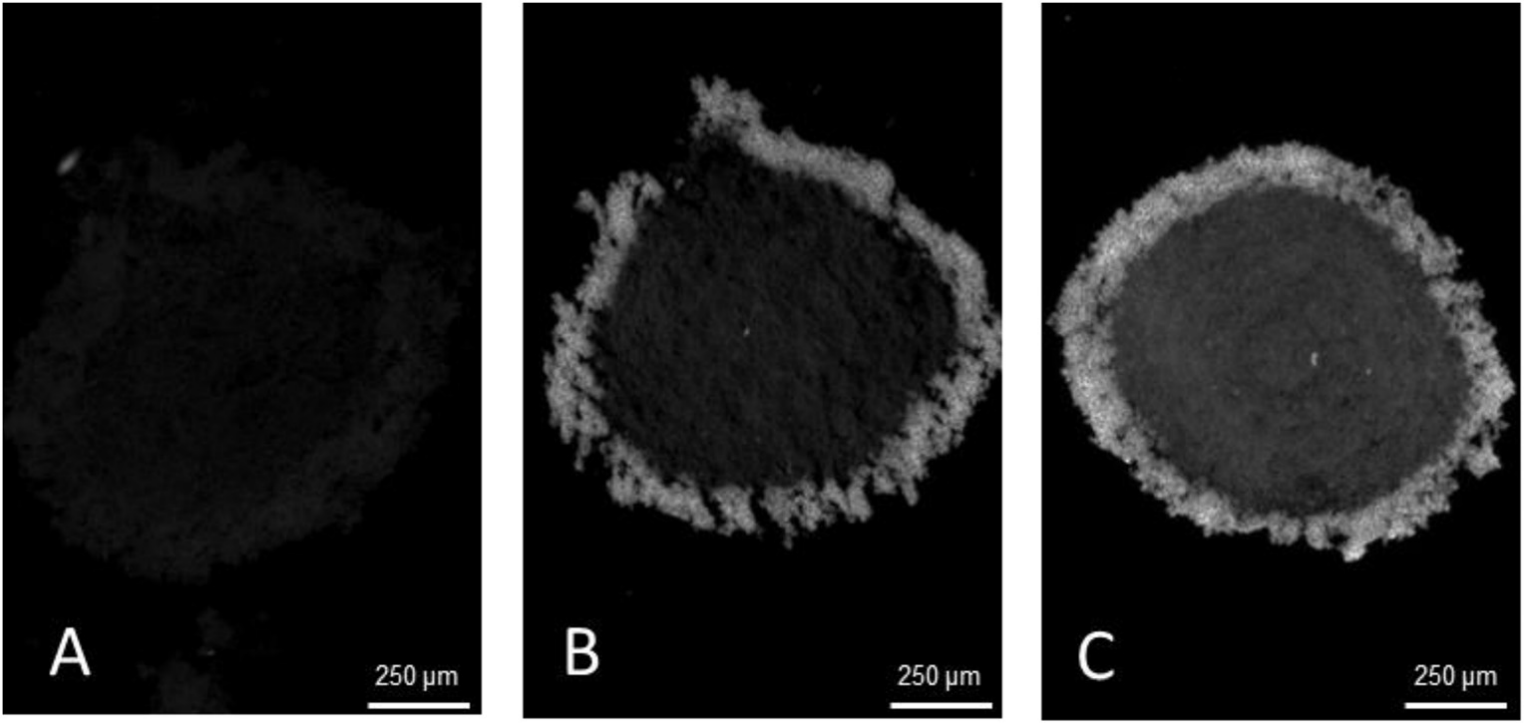
Effect of antigen retrieval treatment on 30 µm sections of 21-day-old HepG2/C3A spheroids fixed with freshly prepared paraformaldehyde and stained for α-SMA. (A) no antigen retrieval, (B) antigen retrieval, 4 min at 92 °C, (C) antigen retrieval, 10 min at 92 °C. All images were captured using 4x objective.

**Fig. 4:**
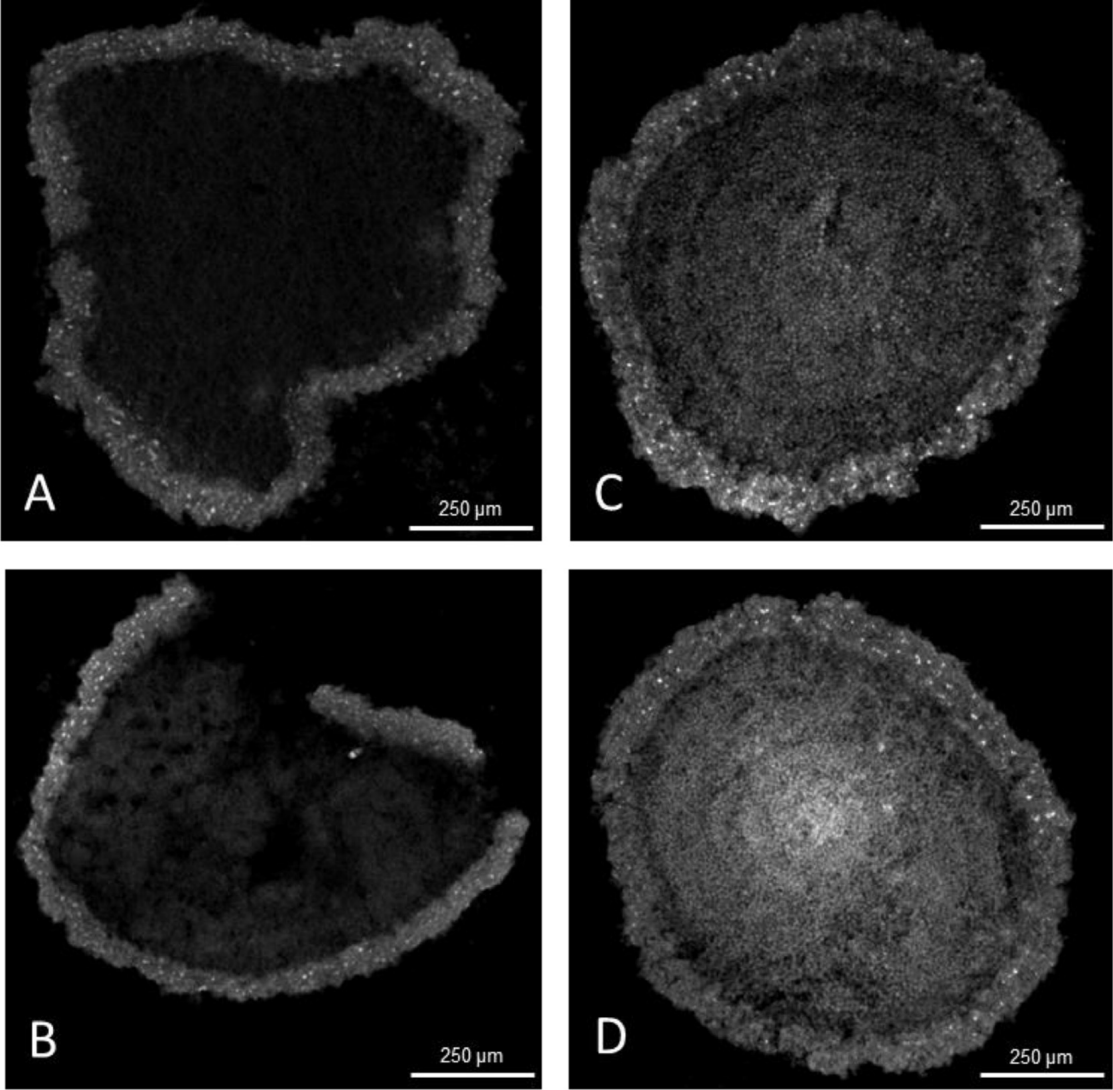
The structural integrity of 30 µm sections of 21-day-old HepG2/C3A spheroids fixed with stabilized paraformaldehyde and stained for α-SMA. Examples of spheroids sections (A, B) damaged and distorted, (C, D) preserved structure. All images were captured using a 4x objective.

The methods presented in this protocol were tested using 21-day-old HepG2/C3A spheroids stained for alpha-smooth muscle actin (α-SMA) (Uniprot Accession Number P62736). 21-day-old HepG2/C3A spheroids are large, approximately 800 to 1000 µm pseudo tissue structures with a viable rim and necrotic core [3,8]. Such spheroids are dense and difficult to analyse using fluorescent light microscopy without sectioning. At the same time, spheroids are fragile and difficult to cut into thin sections without distorting their shape or damaging the internal structures. The presented protocol was compared to a standard cryosectioning and immunostaining protocol [2] and validated using two antigen retrieval conditions with 4 and 10 min heat extraction protocols. The results demonstrate a significant increase in α-SMA staining intensity, with the highest intensity observed after a 10 min heat extraction, Fig. 2, while maintaining spheroid integrity without visible damage. Alterations in tissue embedding and cryosectioning protocols were validated, as previous methods resulted in distorted and broken spheroids, Fig. 3A and B. In contrast, the method outlined in this study improved the quality of cryosections with fewer damaged spheroids and better preservation of their shapes, Fig. 3C and D. As a result of the improved spheroid integrity, thinner sections (10 µm) were obtained, reducing the number of overlapping cell layers, and resulting in clearer and more detailed images, particularly at higher magnifications, Fig. 4.

**Fig. 5:**
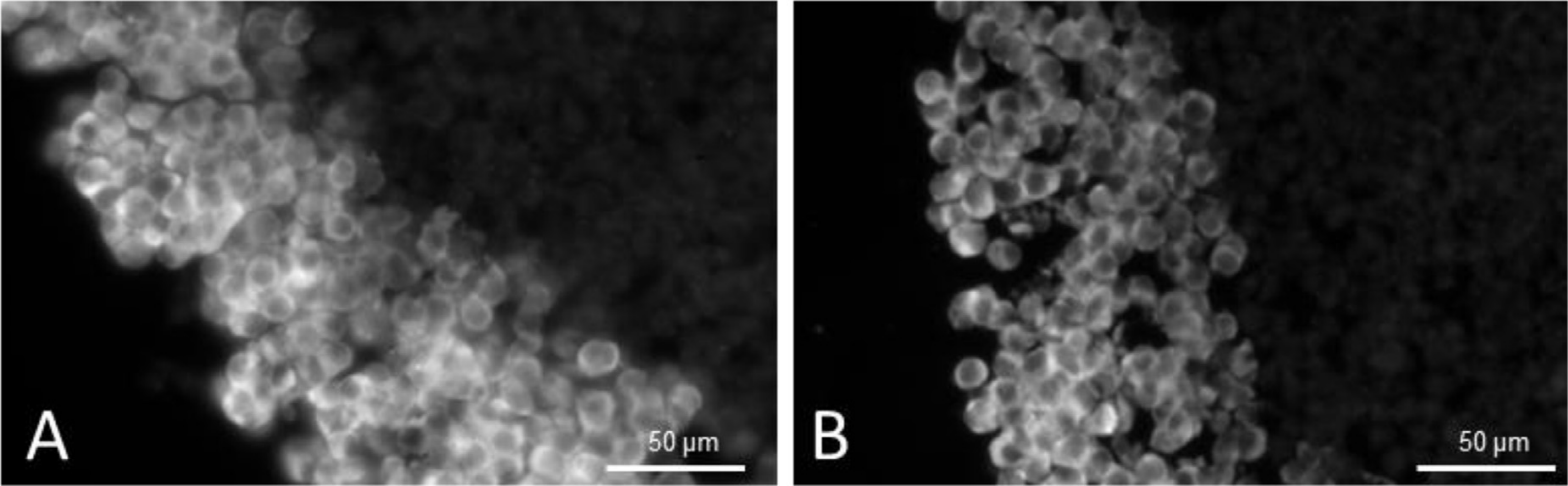
The influence of sectioning thickness on the integrity and image transparency based on 21-day-old HepG2/C3A spheroids stained for α-SMA. (A) 30 µm and (B) 10 µm sections of 21-day-old HepG2/C3A spheroids. Fluorescent staining α-SMA protein. All images were captured using a 20x objective.

This manuscript presents spheroid embedding, cryosectioning, and staining techniques, resulting in the acquisition of immunofluorescent images of superior quality. Notably, this refined protocol has demonstrated a substantial augmentation in fluorescence intensity and preservation of spheroid integrity. These advancements have considerable implications for facilitating more robust experimental hypothesis testing via fluorescence microscopy, as the enhanced visibility and structural integrity of spheroid formations provide a more reliable foundation for analysis and interpretation.

## Ethics statements

This study did not involve human or animal subjects, nor did it collect data from social media platforms.

## CRediT authorship contribution statement

**Claire Charlet-Faure:** Conceptualization, Methodology, Validation, Formal analysis, Investigation, Resources, Writing – original draft, Writing – review & editing, Visualization. **Annemette Præstegaard Thulesen:** Conceptualization, Methodology, Writing – review & editing. **Adelina Rogowska-Wrzesinska:** Conceptualization, Methodology, Resources, Writing – original draft, Writing – review & editing, Visualization, Supervision, Project administration, Funding acquisition.

## Declaration of Competing Interest

The authors declare that they have no known competing financial interests or personal relationships that could have appeared to influence the work reported in this paper.

## Data Availability

Data will be made available on request.

## References

[1] Sakalem M.E., De Sibio M.T., da Costa F., de Oliveira M. (2021). Historical evolution of spheroids and organoids, and possibilities of use in life sciences and medicine. Biotechnol. J..

[2] Wrzesinski K., Frandsen H.S., Calitz C., Gouws C., Korzeniowska B., Fey S.J. (2021). Clinostat 3D cell culture: protocols for the preparation and functional analysis of highly reproducible, large, uniform spheroids and organoids. Methods Mol. Biol..

[3] Frandsen H.S., Stampar M., Vej-Nielsen J.M., Zegura B., Rogowska-Wrzesinska A. (2021). Method to disassemble spheroids into core and rim for downstream applications such as flow cytometry, comet assay, transcriptomics, proteomics, and lipidomics. Methods Mol. Biol..

[4] Ino H. (2003). Antigen retrieval by heating en bloc for pre-fixed frozen material. J. Histochem. Cytochem..

[5] Cizkova K., Flodrova P., Baranova R., Malohlava J., Lacey M., Tauber Z. (2020). Beneficial effect of heat-induced antigen retrieval in immunocytochemical detection of intracellular antigens in alcohol-fixed cell samples. Appl. Immunohistochem Mol. Morphol..

[6] Syrbu S.I., Cohen M.B. (2011). An enhanced antigen-retrieval protocol for immunohistochemical staining of formalin-fixed, paraffin-embedded tissues. Methods Mol. Biol..

[7] Wrzesinski K., Rogowska-Wrzesinska A., Kanlaya R., Borkowski K., Schwammle V., Dai J., Joensen K.E., Wojdyla K., Carvalho V.B., Fey S.J. (2014). The cultural divide: exponential growth in classical 2D and metabolic equilibrium in 3D environments. PLoS One.

[8] Vej-Nielsen J.M., Rogowska-Wrzesinska A. (2021). 3D-ViaFlow: a quantitative viability assay for multicellular spheroids. Methods Mol. Biol..

